# Supplementary material for: Examination of Staphylococcus aureus Prophages Circulating in Egypt
Source: Viruses. 2021 Feb 22;13(2):337. doi: 10.3390/v13020337 (PMC7926752; doi:10.3390/v13020337)
Supplement: Supplementary file 1 [file viruses-13-00337-s001.pdf]

# Examination of *Staphylococcus aureus* prophages circulating in Egypt

**Adriana Ene, Taylor Miller-Ensminger, Carine R. Mores,**
**Silvia Giannattasio-Ferraz, Alan J. Wolfe, Alaa Abouelfetouh and Catherine Putonti**

# Supplementary Materials

## Supplemental Figures

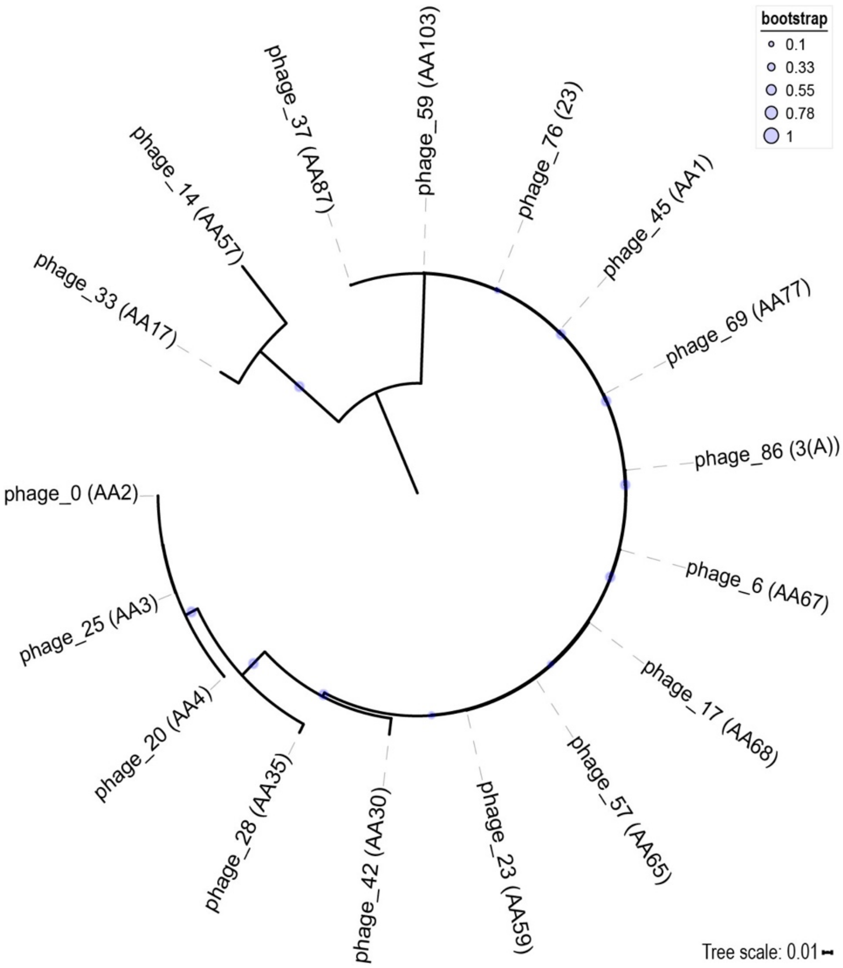


**Supplemental Figure 1. Phylogenetic tree for prophage cluster A prophage sequences.** The *S. aureus* host strain is indicated in parentheses.

## Supplemental Tables

**Table S1. Prophage prediction statistics by strain.**

| Strain | WGS Accession | Isolation Source | # Prophages | | |
|---|---|---|---|---|---|
| | | | Intact | Questionable | Incomplete |
| AA1 | JAEOUR000000000 | BAL | 1 | 0 | 0 |
| AA2 | JAEOUZ000000000 | Blood | 3 | 0 | 0 |
| AA3 | JAEOVE000000000 | BAL | 3 | 0 | 1 |
| AA4 | JAEOVM000000000 | Pus | 3 | 0 | 0 |
| AA5 | JAEOVQ000000000 | Blood | 1 | 1 | 2 |
| AA6 | JAEOVX000000000 | Aspirate | 1 | 1 | 2 |
| AA8 | JAEOWM000000000 | Blood | 1 | 0 | 3 |
| AA13 | JAEOUV000000000 | BAL | 0 | 1 | 3 |
| AA14 | JAEOUW000000000 | Pus | 0 | 1 | 2 |
| AA17 | JAEOUX000000000 | Blood | 2 | 0 | 0 |
| AA18 | JAEOUY000000000 | Pus | 1 | 0 | 2 |
| AA22 | JAEOVA000000000 | Pus | 1 | 2 | 1 |
| AA23 | JAEOVB000000000 | Pus | 0 | 1 | 2 |
| AA27 | JAEOVC000000000 | Pus | 0 | 1 | 1 |
| AA29 | JAEOVD000000000 | Urine | 0 | 1 | 2 |
| AA30 | JAEOVF000000000 | Blood | 2 | 0 | 3 |
| AA31 | JAEOVG000000000 | Pus | 1 | 1 | 2 |
| AA32 | JAEOVH000000000 | Pus | 1 | 0 | 2 |
| AA33 | JAEOVI000000000 | Pus | 1 | 2 | 1 |
| AA35 | JAEOVJ000000000 | Pus | 2 | 0 | 2 |
| AA36 | JAEOVK000000000 | Blood | 2 | 0 | 2 |
| AA39 | JAEOVL000000000 | Aspirate | 1 | 0 | 3 |
| AA41 | JAEOVN000000000 | Blood | 1 | 0 | 2 |
| AA45 | JAEOVO000000000 | Blood | 2 | 0 | 0 |
| AA46 | JAEOVP000000000 | Pus | 0 | 2 | 1 |
| AA51 | JAEOVR000000000 | Pus | 2 | 0 | 1 |
| AA52 | JAEOVS000000000 | Pus | 0 | 1 | 3 |
| AA53 | JAEOVT000000000 | Urine | 1 | 1 | 3 |
| AA55 | JAEOVU000000000 | Pus | 1 | 1 | 2 |
| AA57 | JAEOVV000000000 | Sputum | 2 | 1 | 2 |
| AA59 | JAEOVW000000000 | Pus | 2 | 1 | 1 |
| AA60 | JAEOVY000000000 | MiniBAL | 0 | 1 | 2 |

| Strain | WGS Accession | Isolation Source | # Prophages | | |
|---|---|---|---|---|---|
| | | | Intact | Questionable | Incomplete |
| AA61 | JAEOVZ000000000 | Pus | 0 | 1 | 3 |
| AA62 | JAEOWA000000000 | Pus | 1 | 2 | 1 |
| AA63 | JAEOWB000000000 | MiniBAL | 1 | 1 | 3 |
| AA64 | JAEOWC000000000 | Pus | 1 | 1 | 3 |
| AA65 | JAEOWD000000000 | Urine | 3 | 0 | 1 |
| AA67 | JAEOWE000000000 | Blood | 3 | 0 | 1 |
| AA68 | JAEOWF000000000 | Blood | 3 | 0 | 0 |
| AA69 | JAEOWG000000000 | Blood | 2 | 0 | 1 |
| AA70 | JAEOWH000000000 | Pus | 3 | 0 | 1 |
| AA76 | JAEOWI000000000 | Pus | 1 | 1 | 6 |
| AA77 | JAEOWJ000000000 | Pus | 3 | 0 | 0 |
| AA78 | JAEOWK000000000 | Pus | 1 | 0 | 0 |
| AA79 | JAEOWL000000000 | Sputum | 0 | 2 | 2 |
| AA80 | JAEOWN000000000 | Urine | 1 | 1 | 7 |
| AA87 | JAEOWO000000000 | Pus | 2 | 0 | 1 |
| AA91 | JAEOWP000000000 | Pus | 1 | 1 | 2 |
| AA92 | JAEOWQ000000000 | Pus | 0 | 1 | 1 |
| AA93 | JAEOWR000000000 | Pus | 1 | 1 | 2 |
| AA94 | JAEOWS000000000 | Blood | 1 | 0 | 1 |
| AA95 | JAEOWT000000000 | Pus | 1 | 0 | 1 |
| AA99 | JAEOWU000000000 | Sputum | 1 | 0 | 1 |
| AA101 | JAEOUS000000000 | BAL | 1 | 1 | 2 |
| AA103 | JAEOUT000000000 | Pus | 1 | 0 | 1 |
| AA104 | JAEOUU000000000 | unknown | 1 | 0 | 3 |
| 42 (B) | SUKU00000000 | unknown | 0 | 1 | 2 |
| 48 | SUKQ00000000 | unknown | 1 | 0 | 3 |
| 41 | SUKN00000000 | unknown | 0 | 1 | 4 |
| 6 (A) | SUKY00000000 | unknown | 3 | 0 | 1 |
| 42 | SULE00000000 | unknown | 0 | 1 | 1 |
| 23 | SUKK00000000 | unknown | 2 | 0 | 0 |
| 50 | SUKR00000000 | unknown | 1 | 2 | 2 |
| 3 (B) | SUKH00000000 | unknown | 3 | 0 | 1 |
| 19 | SUKG00000000 | unknown | 0 | 1 | 2 |
| 46 | SUKP00000000 | unknown | 0 | 2 | 2 |
| 40 | SUKM00000000 | unknown | 0 | 2 | 2 |
| 43 | SUKO00000000 | unknown | 2 | 0 | 0 |

| Strain | WGS Accession | Isolation Source | # Prophages | | |
|---|---|---|---|---|---|
| | | | Intact | Questionable | Incomplete |
| 15 | SULC00000000 | unknown | 0 | 0 | 1 |
| 14 | SULB00000000 | unknown | 1 | 1 | 5 |
| 16 | SULD00000000 | unknown | 0 | 0 | 1 |
| 3 (A) | SUKX00000000 | unknown | 3 | 0 | 1 |
| 17 | SWMQ00000000 | unknown | 0 | 0 | 1 |

**Table S2. BLAST hits for the Egyptian *S. aureus* prophages.**

| Prophage ID | Host Strain | Length | Description | Accession # | Query Coverage | % ID |
|---|---|---|---|---|---|---|
| phage_0 | AA2 | 26511 | Staphylococcus phage SA780ruMSSAST101 | NC_048711.1 | 100 | 99.95 |
| phage_1 | AA2 | 29363 | Staphylococcus phage SA97 | KJ716334.1 | 89 | 96.14 |
| phage_2 | AA2 | 43262 | Staphylococcus phage phi7401PVL DNA | AP012341.1 | 100 | 99.94 |
| phage_3 | AA94 | 49880 | Staphylococcus phage 55-3 | KR709303.1 | 66 | 96.3 |
| phage_4 | AA67 | 62863 | Staphylococcus phage Sa2wa_st8 | MK940809.1 | 73 | 99.94 |
| phage_5 | AA67 | 35427 | Staphylococcus phage SA97 | KJ716334.1 | 75 | 97.24 |
| phage_6 | AA67 | 28410 | Staphylococcus phage SAP090B | GQ915271.1 | 81 | 97.73 |
| phage_7 | AA5 | 29682 | Staphylococcus phage B166 | KP893289.1 | 84 | 97.61 |
| phage_8 | AA93 | 16210 | Staphylococcus phage SA13 | JX094501.1 | 92 | 97.84 |
| phage_9 | AA51 | 64168 | Staphylococcus phage SAP090B | GQ915271.1 | 36 | 97.7 |
| phage_10 | AA51 | 31799 | Staphylococcus phage Sa2wa_st8 | MK940809.1 | 99 | 99.97 |
| phage_11 | AA69 | 62863 | Staphylococcus phage Sa2wa_st8 | MK940809.1 | 73 | 99.94 |
| phage_12 | AA69 | 27700 | Staphylococcus phage tp310-1 | EF462197.1 | 79 | 97.34 |
| phage_13 | AA33 | 27203 | Bacteriophage 92 | AY954967.1 | 78 | 97.3 |
| phage_14 | AA57 | 34045 | Staphylococcus phage IME1361_01 | NC_048657.1 | 75 | 98.16 |
| phage_15 | AA57 | 13833 | Bacteriophage 92 | AY954967.1 | 70 | 98.16 |
| phage_16 | AA68 | 62862 | Staphylococcus phage Sa2wa_st8 | MK940809.1 | 73 | 99.94 |
| phage_17 | AA68 | 28102 | Staphylococcus phage SAP090B | GQ915271.1 | 82 | 97.73 |
| phage_18 | AA68 | 31053 | Staphylococcus phage TEM123 | JQ779024.1 | 100 | 99.99 |
| phage_19 | AA4 | 70082 | Staphylococcus phage SA97 | KJ716334.1 | 39 | 96.14 |
| phage_20 | AA4 | 26350 | Staphylococcus phage SA780ruMSSAST101 | NC_048711.1 | 100 | 99.94 |
| phage_21 | AA4 | 43262 | Staphylococcus phage phi7401PVL | AP012341.1 | 100 | 99.94 |
| phage_22 | AA59 | 53697 | Staphylococcus phage Sa2wa_st8 | MK940809.1 | 78 | 99.94 |
| phage_23 | AA59 | 28102 | Staphylococcus phage SAP090B | GQ915271.1 | 82 | 97.73 |

| Prophage ID | Host Strain | Length | Description | Accession # | Query Coverage | % ID |
|---|---|---|---|---|---|---|
| phage_24 | AA3 | 34077 | Staphylococcus phage phi7401PVL DNA | AP012341.1 | 96 | 99.84 |
| phage_25 | AA3 | 26511 | Staphylococcus phage SA780ruMSSAST101 | NC_048711.1 | 100 | 99.95 |
| phage_26 | AA3 | 29363 | Staphylococcus phage SA97 | KJ716334.1 | 89 | 96.14 |
| phage_27 | AA95 | 19196 | Staphylococcus phage 55-3 | KR709303.1 | 65 | 94.91 |
| phage_28 | AA35 | 48171 | Staphylococcus phage SA7 | KY695153.1 | 72 | 99.98 |
| phage_29 | AA35 | 40061 | Staphylococcus phage SA97 | KJ716334.1 | 62 | 97.55 |
| phage_30 | AA32 | 42886 | Staphylococcus phage phiMR25 | AB370205.1 | 61 | 94.7 |
| phage_31 | AA101 | 36605 | Staphylococcus phage phiETA, strain: TY32 | AP008954.1 | 74 | 96.98 |
| phage_32 | AA17 | 62671 | Staphylococcus phage phiSa2wa_st121mssa | MG029518.1 | 68 | 98.57 |
| phage_33 | AA17 | 48021 | Staphylococcus aureus phage SA345ruMSSAST8 | NC_048713.1 | 82 | 99.84 |
| phage_34 | AA18 | 36270 | Staphylococcus phage B166 | KP893289.1 | 71 | 97.47 |
| phage_35 | AA80 | 45707 | Staphylococcus aureus phage phi 12 | AF424782.1 | 60 | 99.79 |
| phage_36 | AA87 | 60588 | Staphylococcus phage phiSa2wa_st121mssa | MG029518.1 | 65 | 99.19 |
| phage_37 | AA87 | 59321 | Staphylococcus phage SAP090B | GQ915271.1 | 39 | 97.74 |
| phage_38 | AA45 | 63945 | Bacteriophage 85 | AY954953.1 | 55 | 96.34 |
| phage_39 | AA45 | 34865 | Staphylococcus phage SA780ruMSSAST101 | NC_048711.1 | 100 | 99.33 |
| phage_40 | AA39 | 54537 | Staphylococcus phage SA7 | KY695153.1 | 63 | 99.97 |
| phage_41 | AA30 | 34597 | Staphylococcus phage phi 11 | AF424781.1 | 76 | 96.55 |
| phage_42 | AA30 | 28121 | Staphylococcus phage tp310-1 | EF462197.1 | 78 | 96.43 |
| phage_43 | AA64 | 22302 | Staphylococcus phage phiETA3, strain: TY32 | AP008954.1 | 91 | 96.98 |
| phage_44 | AA6 | 54537 | Staphylococcus phage SA7 | KY695153.1 | 63 | 99.98 |
| phage_45 | AA1 | 45741 | Staphylococcus phage SAP090B | GQ915271.1 | 50 | 97.73 |
| phage_46 | AA63 | 19610 | Staphylococcus virus B122 | MK290764.1 | 94 | 96.27 |
| phage_47 | AA55 | 30666 | Bacteriophage 92 | AY954967.1 | 70 | 97.3 |
| phage_48 | AA8 | 54537 | Staphylococcus phage SA7 | KY695153.1 | 63 | 99.98 |

| Prophage ID | Host Strain | Length | Description | Accession # | Query Coverage | % ID |
|---|---|---|---|---|---|---|
| phage_49 | AA99 | 49880 | Staphylococcus phage 55-3 | KR709303.1 | 66 | 96.3 |
| phage_50 | AA31 | 24806 | Staphylococcus phage phiETA3, strain: TY32 | AP008954.1 | 92 | 96.89 |
| phage_51 | AA36 | 42543 | Staphylococcus phage SA137ruMSSAST121PVL | MH384261.1 | 86 | 97.82 |
| phage_52 | AA36 | 55314 | Staphylococcus phage SA7 | KY695153.1 | 62 | 99.98 |
| phage_53 | AA53 | 44305 | Staphylococcus aureus phage SA345ruMSSAST8 | NC_048713.1 | 61 | 99.51 |
| phage_54 | AA62 | 36176 | Staphylococcus phage UPMK_2 | MG564297.1 | 70 | 97.1 |
| phage_55 | AA91 | 28172 | Staphylococcus phage phiETA3, strain: TY32 | AP008954.1 | 86 | 96.98 |
| phage_56 | AA65 | 52914 | Staphylococcus phage Sa2wa_st8 | MK940809.1 | 77 | 99.94 |
| phage_57 | AA65 | 28102 | Staphylococcus phage SAP090B | GQ915271.1 | 82 | 97.73 |
| phage_58 | AA65 | 25618 | Staphylococcus phage TEM123 | JQ779024.1 | 100 | 99.99 |
| phage_59 | AA103 | 59360 | Staphylococcus phage SAP090B | GQ915271.1 | 39 | 97.73 |
| phage_60 | AA104 | 54549 | Staphylococcus phage SA7 | KY695153.1 | 63 | 99.93 |
| phage_61 | AA78 | 63535 | Staphylococcus phage SAP090B | GQ915271.1 | 36 | 97.74 |
| phage_62 | AA76 | 47328 | Staphylococcus phage phiMR11 DNA | AB370268.1 | 52 | 95.16 |
| phage_63 | AA22 | 27079 | Bacteriophage 92 | AY954967.1 | 78 | 97.3 |
| phage_64 | AA70 | 81781 | Staphylococcus aureus phage phi 12 | AF424782.1 | 50 | 99.82 |
| phage_65 | AA70 | 50286 | Staphylococcus phage phiETA3, strain: TY32 | AP008954.1 | 69 | 98.3 |
| phage_66 | AA70 | 45450 | Staphylococcus phage SA1014ruMSSAST7 | NC_048710.1 | 68 | 99.17 |
| phage_67 | AA77 | 62863 | Staphylococcus phage Sa2wa_st8 | MK940809.1 | 73 | 99.94 |
| phage_68 | AA77 | 32539 | Staphylococcus phage SA97 | KJ716334.1 | 80 | 97.24 |
| phage_69 | AA77 | 31268 | Staphylococcus phage SAP090B | GQ915271.1 | 73 | 97.73 |
| phage_70 | AA41 | 47772 | Staphylococcus phage phiPVL108 DNA | AB243556.1 | 56 | 98.89 |
| phage_71 | 50 | 14162 | Staphylococcus phage phiETA3, strain: TY32 | AP008954.1 | 99 | 96.92 |
| phage_72 | 48 | 48768 | Staphylococcus phage phiETA3, strain: TY32 | AP008954.1 | 50 | 98.08 |

| Prophage ID | Host Strain | Length | Description | Accession # | Query Coverage | % ID |
|---|---|---|---|---|---|---|
| phage_73 | 43 | 60926 | Staphylococcus phage Sa2wa_st8 | MK940809.1 | 75 | 99.95 |
| phage_74 | 43 | 55555 | Staphylococcus phage SAP090B | GQ915271.1 | 41 | 97.73 |
| phage_75 | 23 | 62863 | Staphylococcus phage Sa2wa_st8 | MK940809.1 | 73 | 99.95 |
| phage_76 | 23 | 59360 | Staphylococcus phage SAP090B | GQ915271.1 | 39 | 97.73 |
| phage_77 | 14 | 45661 | Staphylococcus aureus phage phi 12 | AF424782.1 | 60 | 99.79 |
| phage_78 | 6(A) | 58967 | Staphylococcus phage Sa2wa_st8 | MK940809.1 | 70 | 99.94 |
| phage_79 | 6(A) | 21378 | Staphylococcus phage TEM123 | JQ779024.1 | 100 | 99.99 |
| phage_80 | 6(A) | 28368 | Staphylococcus phage SAP090B | GQ915271.1 | 81 | 97.73 |
| phage_81 | 3(B) | 28368 | Staphylococcus phage SAP090B | GQ915271.1 | 81 | 97.73 |
| phage_82 | 3(B) | 21378 | Staphylococcus phage TEM123 | JQ779024.1 | 100 | 99.99 |
| phage_83 | 3(B) | 58967 | Staphylococcus phage Sa2wa_st8 | MK940809.1 | 70 | 99.94 |
| phage_84 | 3(A) | 58967 | Staphylococcus phage Sa2wa_st8 | MK940809.1 | 70 | 99.94 |
| phage_85 | 3(A) | 24379 | Staphylococcus phage TEM123 | JQ779024.1 | 100 | 99.99 |
| phage_86 | 3(A) | 28496 | Staphylococcus phage SAP090B | GQ915271.1 | 81 | 97.73 |

**Table S3. Coding region statistics of Egyptian *S. aureus* prophages.**

| Prophage ID | Host Strain | Length | # CDS | Antibiotic resistance & virulence factors | Prophage Cluster |
|---|---|---|---|---|---|
| phage_0 | AA2 | 26511 | 26 | *sak; scn* | A |
| phage_1 | AA2 | 29363 | 37 | | G |
| phage_2 | AA2 | 43262 | 58 | *lukS*-PV; *lukF*-PV | E |
| phage_3 | AA94 | 49880 | 70 | | B |
| phage_4 | AA67 | 62863 | 73 | *lukS*-PV; *lukF*-PV | E |
| phage_5 | AA67 | 35427 | 41 | | G |
| phage_6 | AA67 | 28410 | 29 | *sak; scn; sea* | A |
| phage_7 | AA5 | 29682 | 38 | | B |
| phage_8 | AA93 | 16210 | 19 | | - |
| phage_9 | AA51 | 64168 | 72 | *sak; scn; hld; sea; selk; selq* | D |
| phage_10 | AA51 | 31799 | 31 | *lukS*-PV; *lukF*-PV | F |
| phage_11 | AA69 | 62863 | 73 | *lukS*-PV; *lukF*-PV | E |
| phage_12 | AA69 | 27700 | 27 | *sak; scn; sea* | D |
| phage_13 | AA33 | 27203 | 31 | | B |
| phage_14 | AA57 | 34045 | 45 | | A |
| phage_15 | AA57 | 13833 | 17 | | B |
| phage_16 | AA68 | 62862 | 73 | *lukS*-PV; *lukF*-PV | E |
| phage_17 | AA68 | 28102 | 27 | *sak; scn; sea* | A |
| phage_18 | AA68 | 31053 | 40 | | H |
| phage_19 | AA4 | 70082 | 83 | | G |
| phage_20 | AA4 | 26350 | 25 | *sak; scn* | A |
| phage_21 | AA4 | 43262 | 58 | *lukS*-PV; *lukF*-PV | E |
| phage_22 | AA59 | 53697 | 67 | | E |
| phage_23 | AA59 | 28102 | 27 | *sak; scn; sea* | A |
| phage_24 | AA3 | 34077 | 40 | *lukS*-PV; *lukF*-PV | E |
| phage_25 | AA3 | 26511 | 26 | *sak; scn* | A |
| phage_26 | AA3 | 29363 | 37 | | G |
| phage_27 | AA95 | 19196 | 40 | | - |
| phage_28 | AA35 | 48171 | 64 | *sak; scn* | A |
| phage_29 | AA35 | 40061 | 68 | | - |
| phage_30 | AA32 | 42886 | 60 | | H |
| phage_31 | AA101 | 36605 | 47 | | B |
| phage_32 | AA17 | 62671 | 76 | *lukS*-PV; *lukF*-PV | E |
| phage_33 | AA17 | 48021 | 61 | *sak; scn* | A |

| Prophage ID | Host Strain | Length | # CDS | Antibiotic resistance & virulence factors | Prophage Cluster |
|---|---|---|---|---|---|
| phage_34 | AA18 | 36270 | 54 | | C |
| phage_35 | AA80 | 45707 | 47 | *tet(M)* | E |
| phage_36 | AA87 | 60588 | 68 | | F |
| phage_37 | AA87 | 59321 | 67 | *sak; scn; sea; selk; selq* | A |
| phage_38 | AA45 | 63945 | 85 | | H |
| phage_39 | AA45 | 34865 | 43 | *sak; scn* | D |
| phage_40 | AA39 | 54537 | 70 | *sak; scn* | D |
| phage_41 | AA30 | 34597 | 38 | *hlgA; lukE; lukD* | H |
| phage_42 | AA30 | 28121 | 28 | *sak; scn; sea* | A |
| phage_43 | AA64 | 22302 | 28 | | B |
| phage_44 | AA6 | 54537 | 70 | *sak; scn* | D |
| phage_45 | AA1 | 45741 | 53 | *sak; scn; sea; selk; selq* | A |
| phage_46 | AA63 | 19610 | 20 | | C |
| phage_47 | AA55 | 30666 | 36 | | B |
| phage_48 | AA8 | 54537 | 70 | *sak; scn* | D |
| phage_49 | AA99 | 49880 | 70 | | C |
| phage_50 | AA31 | 24806 | 28 | | C |
| phage_51 | AA36 | 42543 | 58 | | F |
| phage_52 | AA36 | 55314 | 74 | *sak; scn* | D |
| phage_53 | AA53 | 44305 | 68 | *sak; chp; scn* | - |
| phage_54 | AA62 | 36176 | 51 | | G |
| phage_55 | AA91 | 28172 | 37 | | C |
| phage_56 | AA65 | 52914 | 65 | | E |
| phage_57 | AA65 | 28102 | 27 | *sak; scn; sea* | A |
| phage_58 | AA65 | 25618 | 29 | | G |
| phage_59 | AA103 | 59360 | 67 | *sak; scn; sea; selk; selq* | A |
| phage_60 | AA104 | 54549 | 70 | *sak; scn* | D |
| phage_61 | AA78 | 63535 | 72 | *sak; scn; hld; sea; selk; selq* | D |
| phage_62 | AA76 | 47328 | 52 | *splA; splB; splC; seg; sei; yent1; yent2; selm; seln; selo; hlgA; lukE; lukD* | G |
| phage_63 | AA22 | 27079 | 30 | | C |
| phage_64 | AA70 | 81781 | 94 | *tet(M)* | F |
| phage_65 | AA70 | 50286 | 72 | | C |
| phage_66 | AA70 | 45450 | 66 | *sak; scn; selp* | D |
| phage_67 | AA77 | 62863 | 73 | *lukS*-PV; *lukF*-PV | E |

| Prophage ID | Host Strain | Length | # CDS | Antibiotic resistance & virulence factors | Prophage Cluster |
|---|---|---|---|---|---|
| phage_68 | AA77 | 32539 | 40 | | H |
| phage_69 | AA77 | 31268 | 35 | *sak; scn; sea* | A |
| phage_70 | AA41 | 47772 | 67 | | F |
| phage_71 | 50 | 14162 | 17 | | B |
| phage_72 | 48 | 48768 | 55 | *splA; splB; splC; splD; splE; splF; hlgA; lukE; lukD* | C |
| phage_73 | 43 | 60926 | 73 | *lukS*-PV; *lukF*-PV | F |
| phage_74 | 43 | 55555 | 65 | *sak; scn; sea; selk; selq* | D |
| phage_75 | 23 | 62863 | 73 | *lukS*-PV; *lukF*-PV | E |
| phage_76 | 23 | 59360 | 67 | *sak; scn; sea; selk; selq* | A |
| phage_77 | 14 | 45661 | 48 | *tet(M)* | E |
| phage_78 | 6(A) | 58967 | 73 | | F |
| phage_79 | 6(A) | 21378 | 26 | | G |
| phage_80 | 6(A) | 28368 | 29 | *sak; scn; sea* | D |
| phage_81 | 3(B) | 28368 | 29 | *sak; scn; sea* | D |
| phage_82 | 3(B) | 21378 | 26 | | G |
| phage_83 | 3(B) | 58967 | 73 | | F |
| phage_84 | 3(A) | 58967 | 73 | | F |
| phage_85 | 3(A) | 24379 | 35 | | G |
| phage_86 | 3(A) | 28496 | 29 | *sak; scn; sea* | A |
